# Advances in SAW Gas Sensors Based on the Condensate-Adsorption Effect

**DOI:** 10.3390/s111211871

**Published:** 2011-12-20

**Authors:** Jiuling Liu, Wen Wang, Shunzhou Li, Minghua Liu, Shitang He

**Affiliations:** Institute of Acoustics, Chinese Academy of Sciences, Beijing 100190, China; E-Mails: liujiuling@mail.ioa.ac.cn (J.L.); wangwenwq@mail.ioa.ac.cn (W.W.); lishunzhou@yahoo.com (S.L.); liuminghua@mail.ioa.ac.cn (M.L.)

**Keywords:** gas sensor, gas chromatography (GC), surface acoustic wave (SAW), threshold detection limit

## Abstract

A surface-acoustic-wave (SAW) gas sensor with a low detection limit and fast response for volatile organic compounds (VOCs) based on the condensate-adsorption effect detection is developed. In this sensor a gas chromatography (GC) column acts as the separator element and a dual-resonator oscillator acts as the detector element. Regarding the surface effective permittivity method, the response mechanism analysis, which relates the condensate-adsorption effect, is performed, leading to the sensor performance prediction prior to fabrication. New designs of SAW resonators, which act as feedback of the oscillator, are devised in order to decrease the insertion loss and to achieve single-mode control, resulting in superior frequency stability of the oscillator. Based on the new phase modulation approach, excellent short-term frequency stability (±3 Hz/s) is achieved with the SAW oscillator by using the 500 MHz dual-port resonator as feedback element. In a sensor experiment investigating formaldehyde detection, the implemented SAW gas sensor exhibits an excellent threshold detection limit as low as 0.38 pg.

## 1. Introduction

**Figure 1.**
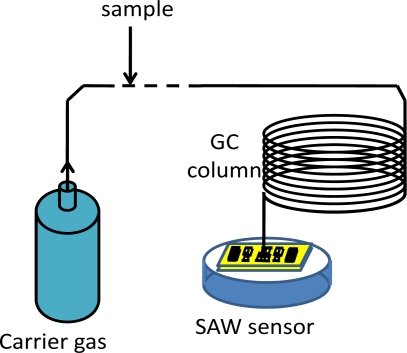
The schematic of the SAW gas sensor based on condensate-adsorption effect.

Portable, accurate and reliable gas sensors are in great demand for monitoring products such as foods, beverages and cosmetics, especially for revealing the presence of volatile organic compounds (VOCs)—like pollutants, explosive materials and drugs. An attractive technique owing to their small size, low cost, high sensitivity and reliability is the use of surface acoustic wave (SAW) gas sensors [1]. A typical SAW gas sensor is composed of a dual-delay line oscillator and a sensitive coating deposited between the input and output interdigital transducer (IDTs) of one delay line [2]. The adsorption of a specific gas by the sensitive coating modulates the phase velocity of the acoustic wave, producing changes at the output of the SAW device, which can be read out by recording the frequency of the oscillator. Many groups have reported successful experiments using such gas sensors [3–9], however, the reported devices still suffer from narrow dynamic range due to the selective scope of chemical films, poor noise rejection and detection limit. To overcome such shortcomings of current SAW gas sensors, a sensor mode using the condensate-adsorption effect was reported, where the target species were condensed on the surface of SAW device by combining a gas chromatography (GC) system [10–16], which resulted in SAW velocity shifts. The schematic and principle of the SAW gas sensor based on the condensate-adsorption effect is depicted in Figure 1.

The sensor system mainly consists of a GC column and a dual-resonator SAW oscillator. The target organic compound samples are first separated when they pass through a temperature programmed GC column. Each compound typically has a different passage velocity in the GC column; this means that the compounds exit the GC column at various characteristic retention times. Identification is accomplished depending on the unique retention time of each compound itself. As each compound sample exits the column, it is condensed onto the surface of the SAW device by controlling the temperature difference between the exit of the GC column and the SAW device, and then the oscillation frequency shift induced by the mass loading and viscous effect from the condensate is used to detect and quantify the target species. Thanks to the high speed of the GC in separating gases (usually only a few seconds to a few minutes), the sensors with GC can be used for real-time monitoring, and for the separation of compounded gases. Furthermore, defects such as inconsistency and instability resulting from sensitive films are avoided.

Many prototypes of SAW gas sensors combined with GCs have been developed over the past several decades, and some commercial products have been applied to VOC detection. Watson *et al.* reported the application of SAW resonators and a quartz capillary column as vapor sensors [12]. Staples *et al.* described the structure and the detection-process of the GC-SAW gas sensor, and many sensor experiments on the detection of volatile and semi-volatile organic compounds, food and beverage by manufacturers were investigated [13–16]. Watson and Staples evaluated the performance of a fast GC equipped with a SAW detector for chemical process measurement, and detection limits of 42 ppb, 10 ppb, 2.5 ppb and 2.5 ppb were obtained for benzene, toluene, ethylbenzene and *o*-xylene, respectively [16]. However, to the best of authors’ knowledge, to the present there is no theoretical model dealing with the sensor response mechanism, which hinders further improvement of the sensor performance, so the first purpose of this paper is to establish a comprehensive and precise theoretical model dealing with the condensate adsorption effect referring to a surface effective permittivity method based on the function of acoustic waves in piezoelectric material and the boundary conditions [17,18], which provides a simple way to describe the SAW propagating velocity shift caused by external mass loading and viscosity effects from the condensate sample. The velocity shift induced by the condensate adsorption effect was depicted, and this allowed prediction of the SAW sensor performance prior to fabrication, especially the threshold detection limit.

Also, it is well known that the sensor performance, especially the threshold detection limit, depends, mainly on the frequency stability of the oscillator. Thus, improvement of the frequency stability is another main topic of SAW gas sensor research. To date, there is almost no literature dealing with frequency stability improvement of the SAW oscillator used for the gas sensor combined with GC. In our work, a new dual-port SAW resonator with the frequency of 500 MHz is fabricated on the same chip as the feedback element of the oscillator. Lower insertion loss, less than 8 dB, Q value higher than 2.2 k, and single resonance mode are observed in the measured data from the fabricated SAW resonator using a network analyzer. A novel phase modulation approach is also applied for the oscillator, which makes the oscillation occur at the lowest insertion loss point, resulting in excellent short-term frequency stability. Thus, superior threshold detection limit is expected, and confirmed with a gas sensor experiment.

## 2. Theoretical Analysis of the Condensation-Adsorption Effect

### 2.1. Condensate-Adsorption Effect

As the operating principle of the GC-SAW gas sensor mentioned in Figure 1, the heated gases from the GC column are condensed onto the surface of the SAW detector at a low temperature as a liquefied layer. Target gases are “adsorbed” onto the surface of the SAW device. Due to the mass loading and viscous property of the condensate itself, the SAW propagation along the piezoelectric substrate is perturbed, causing a velocity shift and attenuation change. The velocity shift induces the oscillation frequency change, which is used for target gas evaluation.

### 2.2. Theoretical Model

**Figure 2.**
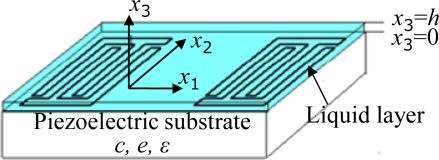
The coordinate system in this study.

In this section, the condensation-adsorption effect is analyzed theoretically by solving the piezoelectric medium equations of motion and surface effective permittivity method. Considering there is an isotropic and piezoelectric medium occupying a half-space (*x_3_* ≤ 0) with interdigital transducer (IDT) about the plane (*x_3_* = 0) and a liquefied condensate adsorbed above (0 ≤ *x_3_* ≤ *h*), as schematically illustrated in Figure 2, the dynamic equations of linear piezoelectricity in a half-space piezoelectric substrate takes the following forms in this coordinate system mentioned in Figure 2:

(1) $$\begin{array}{c} \rho \frac{\partial^{2}u_{i}}{{\partial t}^{2}}-c_{\mathrm{ijkl}}^{E}\frac{\partial^{2}u_{k}}{{\partial x}_{j}{\partial x}_{l}}-e_{\mathrm{ijk}}\frac{\partial^{2}\varphi }{{\partial x}_{j}{\partial x}_{k}}=0\\ e_{\mathrm{jkl}}\frac{\partial^{2}u_{k}}{{\partial x}_{j}{\partial x}_{l}}-\varepsilon_{\mathrm{jk}}\frac{\partial^{2}\varphi }{{\partial x}_{j}{\partial x}_{k}}=0 \end{array}(i,j,k,l=1,2,3)$$

where *x*_1_, *x*_2_, *x*_3_ denote the Cartesian coordinates *x*, *y*, *z* respectively, *E*_ijk_ is the Levi-civita symbol. We denote by *u_i_* the mechanical displacements and by *φ* the electric potential. *c_ijkl_*, *e_kij_*, and *ε*_ij_ stand for the elastic, piezoelectric and dielectric constants, and ρ for the mass density, respectively. The summation convention for repeated tensor indices and the convention that a comma followed by an index denotes partial differentiation with respect to the coordinate associated with the index are adopted. The indices *i*, *j*, *k*, and *l* range from 1 to 3. With the compressed matrix notation [19], the material constants *c_ijkl_* and *e_ijk_* in Equation (1) can be represented by matrices *c_pq_* and *e_ip_*, with the convention that *p*, *q* =1, 2, 3, …, 6. Similarly, the strain tensor *S*_ij_ and the stress tensor *T_ij_* can be represented by *S_p_* and *T_q_*. The first three terms in the left-hand side of Equation (1) are the equation of the motion related to the inertia, the elasticity and piezoelectricity [20].

First, we assume a general solution of Equation (1), the particle displacement and electrical potential, are in the forms of:

(2) $$\begin{array}{cc} u_{i}(x_{1},x_{3})=A_{i}\;\text{exp}\{j\omega t-{\mathrm{jk}}_{s}(x_{1}+\alpha x_{3})\}, & i=1,2,3;\\ \varphi (x_{1},x_{3})=A_{4}\;\text{exp}\{j\omega t-jk_{s}(x_{1}+\alpha x_{3})\} & \end{array}$$

where *k_s_* and *ω* are the wave number in the *x*_1_ direction and the angular frequency, respectively. *α* is a decay constant along the *x*_3_ direction. *A_i_* (*i* =1, 2, 3) and *A*_4_ are wave amplitudes. Substitution of Equation (2) into Equation (1) leads to four linear algebraic equations (Cristoffel equations) for *A_i_* and *A*_4_. Then, for nontrivial solutions of *A_i_* and/or *A*_4_, the determinant of the coefficient matrix of the linear algebraic equations must vanish, and this leads to a polynomial equation of degree eight for *α*. The coefficients of this polynomial equation are generally complex. To ensure the decrease in the displacement *u_i_* and the potential *φ* into the substrate, the generally complex constant *α* must have a negative imaginary part. Thus, we select four eigenvectors with negative imaginary part denoted by *α_n_* (*n* = 1, 2, 3, 4), and the corresponding eigenvectors by $A_{i}^{\left(n\right)}=[A_{1}^{\left(n\right)}\;\;A_{2}^{\left(n\right)}\;\;A_{3}^{\left(n\right)}\;\;A_{4}^{\left(n\right)}]$, *n* = 1, 2, 3, 4. Thus, the general wave solution to Equation (1) in the form of Equation (2) can be written as:

(3) $$\begin{array}{c} u_{i}(x_{1},x_{3})=\sum_{n=1}^{4}A_{i}^{\left(n\right)}C_{n}\;\text{exp}\{-{\mathrm{jk}}_{s}(x_{1}+a_{n}x_{3})\},\;i=1,2,3\\ \varphi (x_{1},x_{3})=\sum_{n=1}^{4}A_{4}^{\left(n\right)}C_{n}\;\text{exp}\{-{\mathrm{jk}}_{s}(x_{1}+a_{n}x_{3})\} \end{array}$$

where *C_n_* (*n* = 1, 2, 3, 4) are the weight factors, and can be determined by the boundary condition.

According to Equation (3) and the piezoelectric constitutive equations, the stress *T*_3*j*_ (*j* = 1, 2, 3) and the electrical displacement component along *x*_3_ direction (*D*_3_) can be obtained in the space of *x*_3_ < 0.

Referring to the Navier-Stokes equations and assuming that the liquefied condensate is a dielectric liquid, the dynamic equation in the liquid layer (0 < *x*_3_ < *h*) takes the following form in this coordinate system mentioned in Figure 2 [21]:

(4) $$\rho \frac{\partial^{2}u_{i}}{\partial^{2}t}=c_{\mathrm{ijkl}}^{}\frac{\partial^{2}u_{k}}{{\partial x}_{l}{\partial x}_{k}}-\frac{2}{3}\mu j\omega \frac{\partial }{{\partial x}_{i}}(\nabla •u)+\mu j\omega \nabla^{2}u_{i}+\mu j\omega \frac{\partial }{{\partial x}_{i}}(\nabla •u),\begin{array}{cc} & i,j,k,l=1,2,3 \end{array}$$

where $\nabla^{2}=\left(\partial^{2}/{\partial x}_{1}^{2}+\partial^{2}/{\partial x}_{2}^{2}+\partial^{2}/{\partial x}_{3}^{2}\right)$ is Laplacian, ∇ • **u** is the divergence of displacement and *μ* is the viscous coefficient. The divergence of displacement is equal zero (∇ • **u** = 0) for incompressible liquid and for incompressible and ideal liquid, the divergence of displacement and viscous coefficients are also equal zero respectively (∇ • **u** = 0, μ =0).

First, we assume a general solution of Equation (4), the particle displacement, is in the form:

(5) $$u^{l}{}_{i}(x_{1},x_{3})=A_{i}^{l}\;\text{exp}\left[j\omega t-j(kx_{1}+a^{l}kx_{3})\right],\begin{array}{cc} & i=1,2,3 \end{array}$$

where the superscript *l* on *u^l^_i_* and *A^l^_i_* differentiates the corresponding parameters from those in Equation (1). Substitution of Equation (5) into Equation (4) leads to three linear algebraic equations (Cristoffel equations) for *A^l^_i_*. Then, for nontrivial solutions of *A_i_*, the determinant of the coefficient matrix of the linear algebraic equations must vanish, and this leads to a polynomial equation of degree six for *α^l^*. All of the generally complex constants *α^l^* are available because of the limit liquid layer and are denoted by *α^l^_n_* (*n* = 1, 2, 3), and the corresponding eigenvectors by $A_{i}^{l(n)}=[\begin{array}{ccc} A_{1}^{l(n)} & A_{2}^{l(n)} & A_{3}^{l(n)} \end{array}]$, *n* = 1, 2, 3. Thus, the general wave solution to Equation (4) in the form of Equation (5) can be written as:

(6) $$u_{i}^{l}\left(x_{1},x_{3}\right)=\sum_{n=1}^{6}A_{i}^{l(n)}C^{l}{}_{n}\;\text{exp}\left[-j\left(kx_{1}+\alpha^{l}{}_{n}kx_{3}\right)\right],\begin{array}{cc} & i=1,2,3 \end{array}$$

According to Equation (6) and the constitutive equations, the stress *T^l^*_3*i*_ (*j* = 1, 2, 3) can be obtained in the space of (0 < *x*_3_ < *h*) as:

(7) $$\begin{array}{l} T_{31}^{l}=\mu (\frac{{\partial v}_{1}}{{\partial x}_{3}}+\frac{{\partial v}_{3}}{{\partial x}_{1}})\\ T_{32}^{l}=\mu (\frac{{\partial v}_{2}}{{\partial x}_{3}}+\frac{{\partial v}_{3}}{{\partial x}_{2}})\\ T_{33}^{l}=\rho v^{2}(\frac{{\partial u}^{l}{}_{1}}{{\partial x}_{1}}+\frac{{\partial u}^{l}{}_{2}}{{\partial x}_{2}}+\frac{{\partial u}^{l}{}_{3}}{{\partial x}_{3}})-\frac{2}{3}\mu (\frac{{\partial v}_{1}}{{\partial x}_{1}}+\frac{{\partial v}_{2}}{{\partial x}_{2}}+\frac{{\partial v}_{3}}{{\partial x}_{3}})+2\mu \frac{{\partial v}_{3}}{{\partial x}_{3}} \end{array}$$

where *v*_1_, *v*_2_ and *v*_3_ are the velocity components along *x*_1_, *x*_2_ and *x*_3_ directions, respectively, ρ and *v* are the density and velocity of the liquid respectively.

Additionally, *φ^l^* and *φ^g^* were introduced to represent the Laplace potential in the space of 0 < *x*_3_ < *h* and in the space of *x*_3_ > *h*, respectively:

(8) $$\begin{array}{l} \varphi^{l}=\varphi^{l}{}_{1}\;\text{exp}(-\mathrm{jk}x_{1}-kx_{3})+\varphi^{l}{}_{2}\;\text{exp}(-\mathrm{jk}x_{1}+kx_{3}),0<x_{3}<h\\ \varphi^{g}=\varphi^{g}{}_{1}\;\text{exp}(-\mathrm{jk}x_{1}-kx_{3}),x_{3}>h \end{array}$$

and they should meet the Laplace equation, which means:

$$\nabla^{2}\varphi^{l}=0,\nabla^{2}\varphi^{g}=0$$

The corresponding electrical displacement component along *x*_3_ direction, *D^l^*_3_, can be written as:

(9) $$\begin{array}{l} D_{3}{}^{l}=-\mathrm{jk}[j\varepsilon_{l}\varphi^{l}{}_{1}\;\text{exp}(-\mathrm{jk}x_{1}-kx_{3})-j\varepsilon_{l}\varphi^{l}{}_{2}\;\text{exp}(-\mathrm{jk}x_{1}+kx_{3})],0<x_{3}<h\\ D_{3}{}^{g}=-\mathrm{jk}[j\varepsilon_{0}\varphi^{g}{}_{1}\;\text{exp}(-\mathrm{jk}x_{1}-kx_{3})],x_{3}>h \end{array}$$

where *ε*_0_ and *ε_l_* are the dielectric constant in the liquid and vacuum respectively.

The solutions of the motion equations should satisfy both the mechanical boundary condition and the electrical boundary condition respectively. The mechanical boundary condition and electrical boundary condition at boundary between the piezoelectric substrate and liquid layer (*x_3_ = 0*) and boundary between liquid layer and vacuum (*x*_3_ = *h*) as schematically illustrated in Figure 2 are:

(10) $$\begin{array}{l} \left\{\begin{array}{c} T_{3j}-T_{3j}^{l}=0\\ u_{j}-u_{j}^{l}=0\\ \varphi -\varphi^{l}=0\\ D_{3}-D_{3}{}^{l}=\sigma (x_{1}) \end{array},j=1,2,3\right\},x_{3}=0\\ \left\{\begin{array}{c} T_{3j}^{l}=0\\ \varphi^{l}-\varphi^{g}=0\\ D_{3}{}^{l}-D_{3}{}^{g}=0 \end{array},j=1,2,3\right\},x_{3}=h \end{array}$$

There are 13 equations in Equation (10), and they can be used to determine 13 unknown terms according to the analyses above (*C_n_*, (*n* = 1, 2, 3, 4); *C^l^_n_*, (*n* = 1–6);, φ^*l*^_1_, φ^*l*^_2_, φ^*g*^_1_). Usually, after solving the Equation (10), the 13 unknown terms ((*C_n_*, (*n* = 1, 2, 3, 4); *C^l^_n_*, (*n* = 1–6); φ^*l*^_1_, φ^*l*^_2_, φ^*g*^_1_)) can be determined. However, due to the inhomogeneity of Equation (10), it is difficult to extract directly the unknown terms. Thus, based on the surface-effective-permittivity method, it is easy to describe the acoustic field in the piezoelectric substrate. The surface effective permittivity constant ε*_s_*(*s*) of the piezoelectric substrate is represented as [22]:

(11) $$\varepsilon_{s}(s)=\frac{\bar{\sigma }\left(s\right)}{2\pi f\left|s\right|\bar{\varphi }\left(s,x_{3}\right)|{}_{x_{3}=0}}$$

Here, *φ̄*(*s*, *x*_3_) is the electrical potential distribution in slowness domain of the piezoelectric substrate; *s* is the slowness, and *s* = 1/*V_s_*, the *V_s_* is the SAW phase velocity. According to Equation (10), *φ*(*s*, *x*_3_) denoted by *σ* can be deduced, and hence the ε*_s_*(*s*) is obtained by substituting into Equation (11).

**Figure 3.**
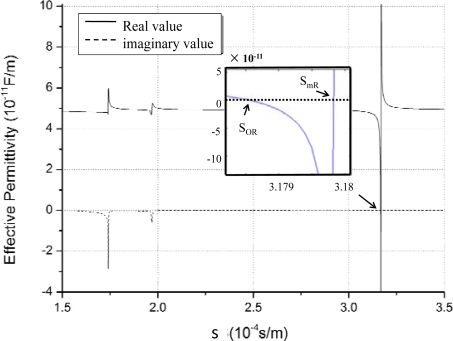
The surface effective dielectric constant ε_s_(s) on ST-X quartz.

Usually, the effective permittivity constant is determined when the material and cut-direction of the crystal substrate, direction of acoustic wave propagation, and normalized thickness of the liquid layer are defined. Using Equations (8–11), the ε_s_(s) on ST-X quartz with Euler angle of (0°, 132.75°, 0°) was obtained as shown in Figure 3. In this case, an ideal liquefied condensate with zero viscosity coefficient on the surface of the quartz, the condensate normalized thickness *kd* (*kd* = *k* × *h*, *k* is the wave number, and *h* is the condensate thickness) of 0.2, and operation frequency of 500 MHz were used. The real line and dotted line described the real part and imaginary part of the *ε_s_*(*s*), respectively. From the picture, there are several discontinuous points, representing different acoustic wave modes. The Rayleigh wave mode is observed at lower acoustic wave velocity (speeds of ∼3.18 × 10^−4^ s/m). There is a zero point and pole point pair in the regime of Rayleigh wave mode with the imaginary part being zero. Where, |ε_s_(S*_mR_*)| → ∞, at pole point of *S_mR_*, represents the slowness of Rayleigh wave propagating along the metallic surface with no attenuation. ε_s_(*S_oR_*) = 0 is observed at zero point of *S_oR_*, and it describes the Rayleigh wave propagating along the free surface with no attenuation. According to the zero and pole points, the phase velocity *V_f_* and *V_m_* in case of free surface and metallic surface coated with condensate can be determined, respectively. To simplify the design procedure, the SAW phase velocity, *V_s_*, can be considered as (*V_f_* + *V_m_*)/2.

Considering the liquid-layer viscosity, the change of the trend of the real part of ε(*s*) is similar to the above result. However, the imaginary part of ε(*s*) is non-zero but negative at the zero point and pole point of Re (ε(*s*)), which denotes the energy attenuation, which in turn was caused by the liquid viscosity.

### 2.3. Numerical Results and Discuss

**Figure 4.**
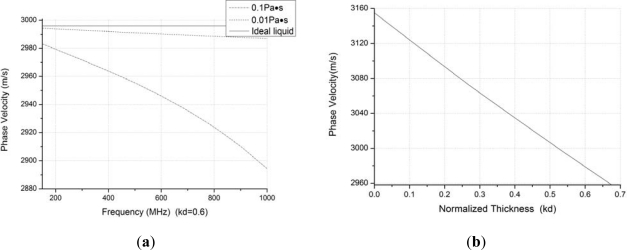
(**a**) Velocity shift from frequency effect and viscosity (**b**) Velocity shift from normalized thickness of condensate.

The piezoelectric material for the SAW sensors analyzed herein is ST-X quartz. Figure 4(a) illustrates the relationships among the velocity shift, operation frequency of sensor, various viscosity coefficient, *μ*, of liquefied condensate (*μ* = 0, *μ* = 0.01 Pa·s, *μ* = 0.1 Pa·s), on the condition that the condensate coating with normalized thickness, *kd*, of 0.6, the density of the condensate ρ of 850 kg/m^3^, and sensing area of 1.5 mm^2^. The higher the operation frequency of the sensor is employed, the larger the velocity shift that will be achieved for the viscous condensate coating with given normalized thickness. This means that a larger sensor response will be achieved when a higher operation frequency is applied. Additionally, the velocity shift increases with an increment of the condensate viscosity coefficient *μ*, as shown in Figure 4(a).

Additionally, the relationship between the phase velocity shift and normalized thickness, *kd*, of condensate is described in case where the Formaldehyde is assumed as the condensate as shown in Figure 4(b). The parameters of density of 820 kg/m^3^, operation frequency of 500 MHz, sensing area of 1.5 mm^2^, and viscosity coefficient assumed as 0.9 × 10^−3^ Pa·s, considering its hydrophilicity, are used to calculate the velocity shift. The velocity shift increases as the normalized thickness of condensate increases, and the velocity shift is approximately proportional to the normalized thickness shift.

Thus, according to the frequency stability description as below, the threshold detection mass of the gas sensor can be deduced by:

(12) $$\Delta m=\frac{\rho sv_{0}{}^{2}\times 3N_{b}}{k_{c}\times 2\pi \times f_{0}{}^{2}}$$

Here, *v*_0_ is the unperturbed SAW phase velocity, *f*_0_ is the operation frequency of the sensor, *N_b_* is the baseline noise of the sensor depending on the frequency stability of the oscillator; *s* is the sensing area, *k_c_* is considered as a scaling constant, which is the quotient of SAW velocity shift Δ*v* caused by the condensate coating and change of normalized condensate thickness Δ(*kd*). Thus, under given adsorption efficiency, *K*, depending on the target specie itself and the experiment condition, the threshold detection limit of the sensor can be evaluated as:

(13) $$m_{\mathrm{DL}}=\frac{\Delta m}{K}=\frac{\rho sv_{0}{}^{2}\times 3N_{b}}{k_{c}\times 2\pi \times f_{0}{}^{2}\times K}$$

From Equation (13), it is obvious that the detection limit is determined by the baseline noise, operation frequency, detection efficiency, and SAW velocity shift caused by the condensate coating. The higher operation frequency, lower baseline noise (superior frequency stability), and larger sensor frequency response (higher *k_c_* value) applied, the lower detection limit would be. Using the sensor for formaldehyde detection as an example, the relationship between the detection limit and adsorption efficiency (*K*) is shown in Table 1, in the case of density of 820 kg/m^3^, baseline noise of 30 Hz, operation frequency of 500 MHz, sensing area of 1.5 mm^2^, and viscosity coefficient assumed as 0.9 × 10^−3^ Pa·s. The *k_c_* value can be obtained by using Equations (1–11) as Figure 4(b). From Table 1, we can find that the detection limit depends mainly on the adsorption efficiency. When increasing the adsorption efficiency, lower detection limit was obtained.

## 3. Technique Realization

In this section, the fabrication process of the SAW sensor is described. It is composed of two dual-port SAW resonators on the same ST-X quartz chip, and corresponding oscillation circuit. From the traditional Tiersten formula in Equation (14) [23], the sensor performance, especially the threshold detection limit, depends mainly on the frequency stability of the oscillator, which acts as the sensor elements:

(14) $$\Delta f/f_{0}=(c_{1}+c_{2}+c_{3})\times f_{0}\times \Delta m/s$$

where Δ*f* is the frequency response towards the gas adsorption with mass loading change of Δ*m*, *f*_0_ is the oscillation frequency, *c*_1_, *c*_2_ and *c*_3_ are the material constants of substrate, and *s* is the area for the liquid layer to cohere. Thus, improvement of the frequency stability is another important topic for the development of a SAW sensor.

Considering the fast sensor response (a few seconds to a minute) of the present gas sensor, the short-term frequency stability of the SAW oscillator acts as a crucial role in the sensor performance evaluation. It is well-known that the short-term frequency stability of oscillator is specified in terms of its output power spectrum *SRF* (*Δf*), as shown in Equation (15) [24,25]:

(15) $$10\text{lg}\;\;S_{\mathrm{RF}}(\Delta f)=10\text{lg}[\frac{\mathrm{GKT}\cdot (\mathrm{NF})\cdot f_{0}^{2}}{Q^{2}P_{0}\Delta f^{2}}]$$

where *G* is the amplifier gain, *NF* is the noise of amplifier, *f_0_* is the central frequency, Δ*f* is offset of *f_0_*, *P_0_* is the saturated output power of the amplifier, *Q* is the Q value of SAW device, *T* is the thermodynamic temperature, and *K* is Boltzmann constant.

It is evident from Equation (15) that for optimum short-term stability the following are required: (a) a SAW device with lower insertion loss; (b) an amplifier with low noise and high power. Thus, design and fabrication of the SAW resonator with low insertion loss and high Q value is one of the main purposes of this paper, and it will be described in the next section.

### 3.1. SAW Resonator

**Figure 5.**
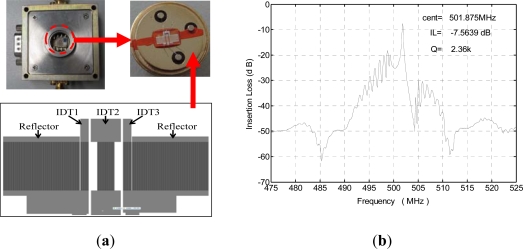
(**a**) The structure of the SAW device, and (**b**) the frequency response of the fabricated device.

From Equation (15), a higher Q value and lower insertion loss of the SAW device will effectively improve the short-term frequency stability of the oscillator [26]. Here, the dual-port resonator structure with three IDTs is used for the SAW device due to its high Q value, low insertion loss, and enough condensation area for gas sensing, as shown in Figure 5(a). By adjusting the distance between one IDT to another, the distance between IDT to grating, and the ratio of IDT period and grating period, only one resonance mode in the frequency bandwidth was expect to be realized. ST-X quartz was chosen as substrate due to its excellent temperature stability. The SAW velocity on ST-X quartz substrate with 300 nm Al IDTs metallization was 3,158 m/s. The operation frequency of the SAW resonator is specifically given by 500 MHz, thus, the wavelength λ of the SAW resonator is given by 6.3 μm. Two dual-port resonator patterns were fabricated on a same ST-X quartz wafer by standard photolithography technique. Each resonator consists of three transducers (IDT1 and IDT3 act as the launching transducers, and IDT2 is the readout transducer) and adjacent shorted grating reflector distributed in each side of the IDT1 and IDT3; the electrode number of the IDT1, IDT2, and IDT3 were 60, 120, and 60, respectively, and the electrode number in shorted grating reflector was set to 400. Spacing between the reflector and IDT was 1.25*λ* (*λ* is the wavelength). Acoustic aperture of 250*λ* and spacing between IDTs of 22.25*λ* were used to accommodate enough condense area for gas sensing. The fabricated SAW device was shown in Figure 5(a). The frequency response S_21_ of the fabricated device was measured with a network analyzer as shown in Figure 5(b). A low insertion loss of 7.6 dB and high Q value of 2,300 were obtained.

### 3.2. SAW Oscillator

Generally, oscillation occurs at the central frequency in the pass band of the SAW devices. However, the central frequency point is not always the frequency point with the lowest insertion loss because of technical issues in the device fabrication. To improve the short-term frequency stability of the oscillator, the oscillation in frequency point with the lowest insertion loss of the SAW devices is advised here by adjusting the phase shifter.

**Figure 6.**
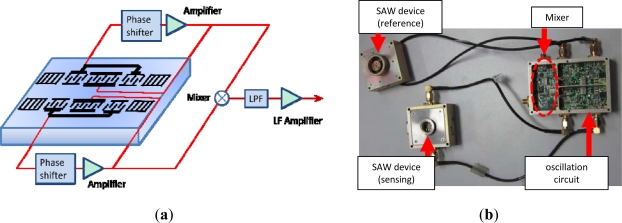
(**a**) Schematic and principle of the SAW oscillator, (**b**) the PCB with the SAW sensor.

Then, using the fabricated SAW devices as feedback, a 500 MHz SAW oscillator is implemented with an oscillator circuit composed of discrete elements (amplifier, phase shifter, mixer and LPF, *etc*.) on a printed circuit board (PCB) as shown in Figure 6(b). The working principle of the oscillator was as depicted in Figure 6(a). The output of the amplifier is mixed in order to obtain a differential frequency in the MHz range. This technique allows us to reduce the influence of the thermal expansion of the substrate and to use simple low-frequency counters. The output of the oscillator is connected to a programmable frequency counter which can be used for an analog indicator or a chart recorder.

**Figure 7.**
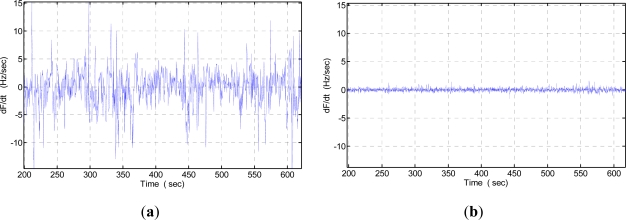
Frequency stability testing of oscillator modulated at the frequency point of (**a**) central frequency point, and (**b**) at the lowest insertion loss point.

An experiment was performed to evaluate the frequency stability of the fabricated SAW oscillator using the programmable frequency counter under constant testing temperature (25 °C) and 70% RH. To demonstrate the short term frequency stability (seconds), a partial differentiation is used. It shows the frequency shift per second. The testing condition is 25 °C temperature. Then, the oscillation is modulated at the frequency point with the lowest insertion loss by a strategic phase modulation, in which a low pass filter acts as the phase shifter, and the phase modulation is accomplished by adjusting the inductor or capacitor values. The measured short-term frequency stability (frequency shift in seconds) is shown in Figure 7(b). Excellent short-term frequency stability of ∼±3 Hz per second is obtained, much better than that of the previous oscillator with oscillation at the operation frequency point, in which, weak short-term frequency stability of ±15 Hz were observed as shown in Figure 7(a).

## 4. Sensor Experiments

Based on the theoretical analysis on response mechanism mentioned before and the optimized design on the SAW oscillation, a SAW sensor based on condensation-adsorption effect with operation frequency of 500 MHz is implemented for VOCs sensing. The gas sensing setup, depicted in Figure 1, comprises a carrier gas (N_2_), VOCs sample, valve, GC column, SAW detector, temperature-control system and a programmable frequency counter connected to a computer. The frequency signal was processed with the software.

The experimental operation of the present SAW sensor is divided into a two-step process. The first step is sample preparation and organic compound collection. Once the sampling is completed, the collected organic compounds are injected into the carrier gas flow leading to the GC column. Then the compounds are separated by the GC column and detected by the SAW sensor. Here, the temperature of the GC column is 200 °C, the temperature of the SAW device is 25 °C, and nitrogen works as the carrier gas. Additionally, the surface of the SAW device was cleaned by the carrier gas prior to the sensor experiment. Then, the residue on the surface of the SAW device after gas sensing experiments can be cleaned up by heating to 150 °C.

Also, to sharpen the response peaks in experimental signal processing, the partial differential was applied on the output frequency signal to test time. Thus, in the following pictures, the vertical coordinate refers to the partial differential values (d*f*/dt), whereas the horizontal ordinate represents the test time. Thus, the real frequency response in VOCs sensing was the area of response peak in the pictures.

**Figure 8.**
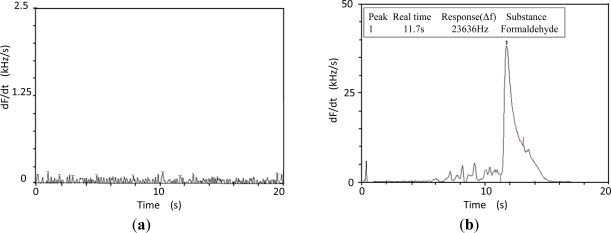
Sensor responses to different inputs (**a**) no input, (**b**) 0.1 ng derived formaldehyde.

Next, the fabricated SAW sensor was used for the detection of formaldehyde. To improve the adsorption, a derivatization was applied. The baseline noise of the sensor system was measured as Figure 8(a), so very low noise was observed. Then, 0.1 ng formaldehyde was derived prior to injection in the GC column by the carrier gas. The induced sensor response is depicted in Figure 8, a large and clear frequency response of over 23 kHz was observed in 0.1 ng formaldehyde detection.

According to the International Union of Pure and Applied Chemistry (IUPAC), detection limits are calculated as the lowest concentration of an analyte giving a signal of at least three-times the short term frequency stability of the sensor system. To keep a stable detection limit evaluation, the baseline noise of the gas sensor was considered as 5 times the short term stability of the oscillator (∼30 Hz). Thus, the detection limit of the sensor for formaldehyde detection is evaluated as 0.38 pg, close to the calculated value of 0.3 pg shown in Table 1, in case of the adsorption efficiency near 1, which is consistent with the hydrophilic nature of the formaldehyde.

## 5. Conclusions

This paper reports some work on SAW gas sensors based on the condensation-adsorption effect relating to the response mechanism, design and fabrication of the SAW oscillator. The response mechanism of the gas sensor is analyzed theoretically to predict the sensor performance by referring to the surface effective permittivity method. A 500 MHz SAW two-port resonator with three IDTs structure is fabricated as the feedback element of the oscillator, which exhibits high Q value, low insertion loss, and enough condensing area for gas sensing. By applying a new phase modulation method, excellent short-term frequency stability (±3 Hz) is measured for the presented SAW oscillator. By using the fabricated SAW oscillator as the sensor element, the proposed SAW gas sensor is used for VOCs detection; superior performance like a lower threshold detection limit of approximately 0.38 pg for formaldehyde is observed experimentally.

## Figures and Tables

**Table 1.** The calculated detection limit for formaldehyde for various adsorption efficiencies.

**Adsorption efficiency *K***	10%	40%	70%	100%
**Threshold detection limit m_DL_ (*pg*)**	3.0	0.75	0.43	0.3
